# La Covid-19 en Côte d'Ivoire (mars 2020 - avril 2021) une année sous le sceau du coronavirus

**DOI:** 10.48327/MTSIMAGAZINE.N1.2021.102

**Published:** 2021-04-28

**Authors:** J.-M. Milleliri, D. Coulibaly, F. Lamontagne

**Affiliations:** 1 GISPE, 82 bd Tellène, 13007 Marseille, France; 2 INHP, BP V14, Abidjan, Côte d'Ivoire

**Keywords:** Covid-19, Coronavirus, Épidémiologie, Côte d'Ivoire, Afrique, Covid-19, Coronavirus, Epidemiology, Ivory Coast, Africa

## Abstract

Après un an d’épidémie de coronavirus, la Côte d'Ivoire termine une troisième vague de Covid-19. Si l’épidémie a été confinée majoritairement dans le Grand Abidjan, notamment grâce aux mesures d'isolement imposée à la capitale économique ivoirienne, l'impact de la crise sanitaire a été néanmoins marqué. À l'instar d'autres pays d'Afrique de l'ouest, la Côte d'Ivoire n'a pas connu le tsunami épidémique que certains prévoyaient en mars 2020, mais plus de 45 000 cas et près de 300 décès ont été notifiés, tout en sachant que ces chiffres sous-estiment la réalité épidémiologique. Avec l'arrivée de la vaccination, la Côte d'Ivoire espère pouvoir contrôler l’épidémie mais la possible circulation de variants notamment sud-africains et les difficultés d'approvisionnement en doses vaccinales sont autant de défis que devront relever les autorités sanitaires ivoiriennes. La résilience des populations a été importante durant cette crise, illustrant la capacité des Ivoiriens à résister à l'impact de cette crise.

## Abstract (en)

After a year of coronavirus epidemic, Côte d'Ivoire is completing a third wave of Covid-19. Although the epidemic has been confined mainly to Greater Abidjan, thanks in particular to the isolation measures imposed on the Ivorian economic capital, the impact of the health crisis has nevertheless been marked. Like other West African countries, Côte d'Ivoire did not experience the epidemic tsunami that some predicted in March 2020, but more than 45 000 cases and nearly 300 deaths have been reported, although these figures underestimate the epidemiological reality. With the advent of vaccination, Côte d'Ivoire hopes to control the epidemic, but the possible circulation of variants, particularly South African variants, and the difficulties in obtaining vaccine doses are challenges that the Ivorian health authorities will have to overcome. The resilience of the population has been significant during this crisis, illustrating the ability of Ivorians to withstand the impact of this crisis.

## Introduction

La Côte d'Ivoire, est un pays d'une superficie de 322 462 km^2^, dont la population est estimée en 2020 à plus de 25 millions d'habitants. Le taux de croissance démographique annuel est évalué à 2,6 % avec un taux d'urbanisation de 51,6 %. La capitale économique, Abidjan compte plus de 5 millions d'habitants. L'espérance de vie à la naissance est de 56,8 ans. La population est jeune, les moins de 15 ans représentant près de 42 % de la population et l'ensemble des moins de 35 ans plus de 77 %.

Le premier cas de Covid-19 a été notifié le 11 mars 2020 chez un ressortissant ivoirien arrivant d'Italie.

Au 30 septembre 2020, la Côte d'ivoire avait notifié 19 724 cas de Covid-19 et 120 décès.

L’épidémie a connu une croissance notable jusqu'en juillet selon la chronologie suivante: 1er cas le 11 mars, 100 cas le 28 mars, 1 000 cas le 24 avril, 5 000 cas le 15 juin et 10 000 cas le 4 juillet.

Au 30 septembre 2020, 19 291 malades ont été déclarés guéris soit un taux de guérison de 98,4 %. A cette date, il demeurait 313 cas actifs.

Plus d'un an après le début de l’épidémie, au gré de trois vagues épidémiques dont la dernière est en train de se terminer, la Côte d'Ivoire avait enregistré au 17 avril 2021, 45 560 cas et 274 décès soit un taux de létalité de 0,6 %.

Le démarrage de la vaccination en mars 2021, combiné à la poursuite des mesures barrières renforcées, est un espoir de pouvoir contrôler l’épidémie.

Un an après le début de l’épidémie ivoirienne, cet article fait le point des données épidémiologiques qui ont marqué la crise sanitaire en Côte d'Ivoire, et analyse les mesures mises en place par les autorités nationales pour y faire face.

## Évolution des mesures de contrôle: 4 mois de confinement du grand abidjan^1^

Force est de constater que tout au long de l’évolution de la crise sanitaire, le gouvernement ivoirien a su adapter les règles de prévention à la dynamique de l’épidémie avec des mesures contraignantes, des relâchements ponctuels et des reprises de certaines mesures.

Ainsi, dès le 16 mars 2020, soit cinq jours après l'identification du 1^er^ cas, le Conseil National de Sécurité a édicté 13 mesures contraignantes pour limiter l'extension de l’épidémie parmi lesquelles la suspension pour 15 jours renouvelables de l'entrée en Côte d'Ivoire des voyageurs non ivoiriens en provenance des pays ayant plus de 100 cas confirmés de Covid-19. En plus des contrôles sanitaires aux frontières, est décrétée la fermeture de tous les établissements d'enseignement pour 30 jours, l'interdiction de rassemblement de plus de 50 personnes et la suspension de tout événement sportif ou culturel. L'application des mesures barrières, y compris le port du masque, est placée en avant dans la stratégie de lutte contre l’épidémie.

Le 22 mars, le gouvernement ivoirien publie son plan de riposte national avec la création à la Primature d'un cadre de gestion des urgences, de suivi et d’évaluation de la matrice d'actions et des mesures de prévention dans l'optique de renforcer la coordination multisectorielle pour la riposte du pays. Des comités techniques opérationnels sont mis en place par le Ministère de la Santé pour la prise en charge des cas, le suivi des contacts, la surveillance épidémiologique, la communication préventive et le déploiement de moyens de protection (masques, site de lavage des mains). Le budget du Plan de riposte contre la Covid-19 s’élève à près de

96 milliards de FCFA (plus de 146 millions d'Euros) avec un Fonds de solidarité de 20 milliards de francs CFA (plus de 30 millions d'Euros) pour la distribution de vivres et d’équipements essentiels aux populations vulnérables.

Le lendemain, le 23 mars, dans une adresse à la Nation, le président de la République, Alassane Dramane Ouattara, déclare l’état d'urgence sanitaire avec des mesures additionnelles contraignantes dont l'instauration d'un couvre-feu de 21h00 à 05h00 du matin et surtout l'interdiction des déplacements non autorisés entre le Grand Abidjan et l'intérieur du pays. Dès lors confinée, la population abidjanaise va vivre l’épidémie en vase-clos. L’épidémie devient essentiellement une épidémie urbaine: 95 % des cas sont déclarés dans le Grand Abidjan.

Le 9 avril 2020, des mesures de renforcement des dispositions sont mises en place comme l'obligation du port de masques dans le Grand Abidjan, le confinement obligatoire à domicile de toutes les personnes fragiles, la mise en œuvre du télétravail, la réduction du nombre de passagers dans les véhicules de transport en commun. Parallèlement, sont installés à Abidjan 13 centres de prélèvements pour les tests PCR sur les 45 centres programmés pour l'ensemble du territoire.

Le 20 avril, le Premier ministre, feu Amadou Gon Coulibaly, annonce la mise en place d'un Plan de soutien économique, social et humanitaire de 1 700 milliards de FCFA (près de 2,6 milliards d'Euros) pour soutenir l'outil de production et maintenir les emplois dont 530 milliards (près de 808 millions d'Euros) financés par le Fonds Monétaire International.

Le 24 avril, le couvre-feu est prorogé jusqu'au 8 mai sur l'ensemble du territoire, avant d’être levé le 15 mai. Néanmoins, le confinement du Grand Abidjan est prolongé jusqu'au 31 mai mais les établissements d'enseignement sont rouverts à compter du 25 mai. À cette date, la Côte d'Ivoire a notifié 2 376 cas et 30 décès. Le 11 juin - alors que depuis le 5 juin le nombre de cas hebdomadaires a dépassé les 1 100 cas, tandis qu'il était inférieur à 400 les deux semaines précédentes - le Conseil national de sécurité prolonge l’état d'urgence jusqu'au 30 juin, le maintien de l'isolement et le confinement des populations du Grand Abidjan. Le 15 juin, le nombre de cas notifiés atteint le chiffre de 5 000. Le nombre maximum de personnes autorisées à se réunir, mesure qui avait été atténuée, est de nouveau réduit de 200 à 50 personnes.

Ce confinement du Grand Abidjan, interdisant sauf exception le déplacement des populations en dehors de la capitale économique, conduit à donner à l’épidémie un caractère limité géographiquement. En effet, même si quelques cas sont notifiés dans les provinces (mais ces cas sont le plus souvent rapatriés sur Abidjan), les cas notifiés à Abidjan représentent 95 % des cas nationaux. D'ailleurs, ce confinement urbain est prolongé jusqu'au 15 juillet, date à laquelle il est levé ayant ainsi durant quatre mois isolé les populations abidjanaises de celles des provinces. Durant quatre mois, les bars, boîtes de nuit, cinémas et lieux de spectacle ont également été fermés.

Le 1^er^ juillet 2020, la Côte d'Ivoire a rouvert son espace aérien fermé depuis mars aux vols internationaux et tous les passagers arrivant sont soumis à un contrôle sanitaire systématique, lequel sera renforcé en août par l'obligation de présenter une attestation de test PCR négatif de moins de 72 heures à l'entrée sur le territoire national (délai élargi à 7 jours le 22 septembre au moment où le gouvernement met en place une procédure de déclaration de réalisation de test PCR obligatoire pour les passagers quittant la Côte d'Ivoire).

**Figure 1:**
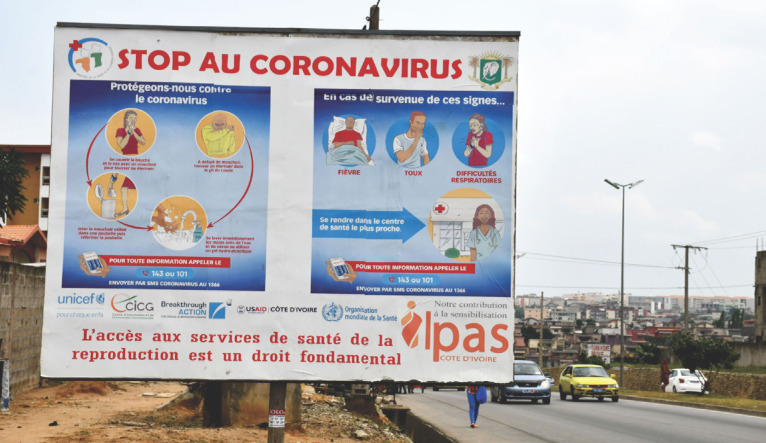
Panneau de sensibilisation pour la lutte contre la Covid-19. Quartier Cocody, Abidjan, août 2020. ©GISPE Awareness panel for the fight against Covid-19. Cocody neighborhood, Abidjan, August 2020. ©GISPE

À partir du mois d'octobre 2020, fort de données épidémiologiques rassurantes, les mesures contraignantes n'ont plus été d'actualité en Côte d'Ivoire. La plupart des panneaux de sensibilisation pour la lutte contre la Covid-19 (Fig. 1) – qui jusqu'alors étaient en place un peu partout à Abidjan – ont été remplacés par ceux lançant la campagne présidentielle ivoirienne. Dans les rues, le pourcentage de personnes portant des masques a chuté, et seules les grandes surfaces commerciales d'enseignes souvent internationales ont continué à mettre en place des mesures de prévention rigoureuses.

Finalement ce sont les mesures de fermeture de frontières imposées par certains pays dont la France, le 31 janvier 2021, qui ont peut-être eu le plus d'impact contraignant sur une partie de la population ivoirienne habituée à voyager. De son côté, les autorités ivoiriennes ont réduit de deux jours la validité d'un résultat de test PCR négatif pour pénétrer sur son territoire, ce délai passant de 7 à 5 jours.

Fort de son expérience passée à contrôler différentes épidémies et notamment celle de la maladie à virus Ebola (MVE) qui a sévi en Afrique de l'ouest en 2013-2015, la Côte d'Ivoire a su mettre en place un ensemble de mesures ayant permis de limiter l'impact de la crise sanitaire. Ainsi, transférant à la gestion de l’épidémie à coronavirus les connaissances acquises lors de l’épidémie à MVE, des plans d'actions ont pu être mis en place très précocement sous forme de cadre logique d'intervention, des programmes de sensibilisation du public et de mobilisation communautaire, une surveillance épidémiologique avec des formations au niveau des districts et l'appui de l'OMS et d'ONG internationale [1, 2].

## Évolution de l’épidémie: trois vagues en une année

Au 11 mars 2021, soit un an après que la Côte d'Ivoire ait notifié son premier cas, au moment où l'OMS déclarait l’état d'urgence sanitaire mondial et classait l’épidémie en pandémie, force est de constater que l’épidémie ivoirienne est restée sur un mode actif mais relativement modéré. L’épidémie ivoirienne n'a pas connu le niveau de certains pays africains comme par exemple l'Afrique du Sud mais en Côte d'Ivoire hormis à la fin de l'année 2020 – laissant en espérer presque un contrôle – l’épidémie est restée active, bien que majoritairement située à Abidjan. Cette concentration des cas dans la capitale économique ivoirienne permet de caractériser l’épidémie comme une épidémie urbaine [3], son extension en province ayant été sinon contrôlée, tout au moins limitée à 5 % des cas.

Ainsi, sur le plan épidémiologique, il est possible de situer le premier pic épidémique dans le courant du mois de juillet. Les taux d'attaque hebdomadaire pour 100 000 habitants pour la ville d'Abidjan, après un premier pic dans la semaine du 15 au 21 juin (37,73), ont connu un plateau supérieur à 25 pour 100 000 jusqu'au 6 juillet, puis un pic du 6 au 12 juillet à 32,80 pour 100 000. Les quartiers d'Abidjan ont été touchés de façons différentes: deux communes de la capitale ont constitué des clusters importants: Marcory-Treichville et Cocody-Bingerville. Ces deux communes ont connu aux mêmes périodes des taux d'attaque hebdomadaire de plus de 100 pour 100 000 habitants. Ces deux communes ont regroupé à elles seules 60 % des cas notifiés. Ainsi, si l'on considère au 30 septembre que 95 % des cas ont été notifiés sur Abidjan ce sont plus de 18 730 cas qui sont survenus dans la capitale économique ivoirienne (19 724 x 0,95). Les taux d'attaque cumulés pour 100 000 habitants au 30 septembre 2020 ont été respectivement de 78,58 pour le pays et 367,4 pour Abidjan.

Au 30 septembre, sur les 113 districts sanitaires que compte la Côte d'Ivoire, 66 avaient notifié au moins un cas depuis le début de l’épidémie. Au 17 avril 2021 ce sont 87 districts sanitaires qui avaient été touchés. Les 10 districts de la ville d'Abidjan dans lesquels ont été notifiés 95 % des cas nationaux étaient encore tous actifs à cette date.

Après la levée de mesures sanitaires strictes dont le déconfinement du Grand Abidjan et la réouverture des lieux socio-culturels ou éducatifs, un second pic épidémique est survenu en janvier 2021. Ce second pic est apparu en janvier sans doute à la faveur de plusieurs facteurs: effet des rassemblements de la campagne électorale présidentielle, rencontres familiales et festives des fêtes de fin d'année, mouvements de voyageurs se rendant en Côte d'Ivoire pour les fêtes, relâchement de mesures barrières des populations dans certains quartiers… Il est à noter que peu de manifestations populaires ont eu lieu en Côte d'Ivoire pour s'opposer aux mesures contraignantes de prévention hormis en avril 2020 la mise à sac d'un centre de dépistage érigé dans le quartier de Yopougon. Si lors du premier pic l'incidence moyenne par semaine avait culminé à 300 cas par jour le 5 juillet, cette incidence est remontée à 275 cas par jour fin janvier 2021, après avoir connu une baisse jusqu’à 11 cas par jour à la mi-novembre 2020.

Ainsi, entre le 4 janvier 2021 (date de reprise de l’épidémie) et le 26 février 2021 (date du début de la troisième vague), le nombre de nouveaux cas s'est élevé à 19 535 soit une moyenne de 355 cas par jour. Si une accalmie a pu être observée entre le 16 février et le 2 mars 2021 (moyenne quotidienne de 78 cas par jour), l’épidémie a connu dès la fin du mois de février une reprise avec une moyenne quotidienne de 370 cas sur les 11 premiers jours de mars.

**Figure 2:**
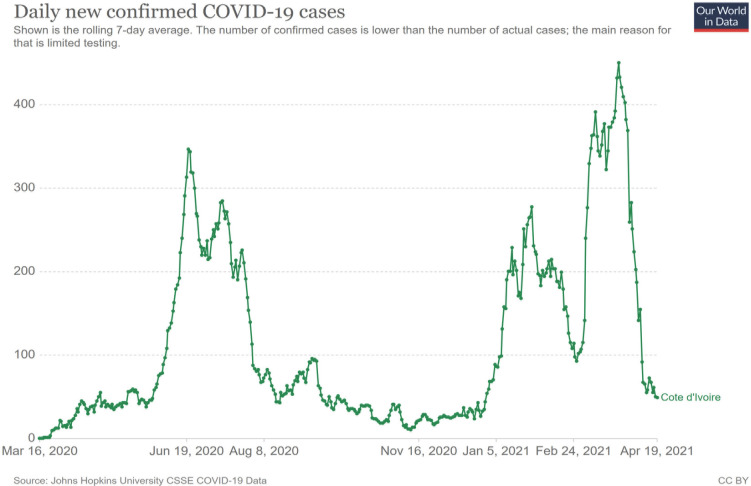
Courbe de l’évolution du nombre de cas quotidiens de Covid-19 (en moyenne de suivi hebdomadaire) entre le 16 mars 2020 et le 19 avril 2021 Covid-19 daily case trend curve (weekly follow-up average) between March 16, 2020 and April 19, 2021 Source: https://ourworldindata.org/covid-cases

La troisième vague épidémique en 2021 a ainsi été marquée en Côte d'Ivoire par un pic d'incidence le 25 mars de 767 cas (moyenne de 450 cas par jour sur les 7 derniers jours), soit 29 cas par million d'habitants. À partir de cette date, l’épidémie a décru à une incidence le 19 avril de 49 cas quotidiens soit 1,86 cas par million d'habitants (Fig. 2). Cette troisième vague a été plus importante que les deux premières. La première vague dont la croissance s'est étalée entre le 25 mai et le 12 juillet (pic) a enregistré 10 343 cas (soit une moyenne de 215 cas quotidien), la seconde entre le 28 décembre 2020 et le 29 janvier 2021 en a enregistré 5 518 (soit une moyenne de 184 cas quotidien), tandis que la dernière dont la croissance s'est étalée entre le 26 février et le pic du 25 mars a enregistré 9 596 cas (soit une moyenne quotidienne de 330 cas quotidien).

La notification des cas de Covid-19 en Côte d'Ivoire a donc été majoritairement rapportée dans le Grand Abidjan qui enregistrait à la date du 17 avril, 43 115 cas sur les 45 560 cas nationaux, soit près de 95 % des cas. Depuis le début de l’épidémie, 572 731 échantillons nasopharyngés ont été testés en PCR avec un taux global de positivité de 8 % à la date du 17 avril (45 560/572 731). Mais, de nombreux cas n'ont pas été diagnostiqués soit qu'il s'est agi de cas asymptomatiques ou paucisymptomatiques, soit que le diagnostic n'ait pas été porté en raison de la difficulté d'accès à un test PCR en dehors des centres à Abidjan ou Bouaké. Ainsi, un suivi de sérologies réalisé chez des employés d'une société minière en Côte d'Ivoire [4] a révélé une séroprévalence du SRAS-CoV-2 de 35 % aussi élevée chez les travailleurs des mines d'or en province que chez le personnel administratif vivant à Abidjan. Ce taux de séroprévalence élevé, relevé au sein des employés de cette société, a été atteint avant juillet 2020 et est resté stable après la première vague pandémique, entre juillet et octobre 2020. Cette épidémie silencieuse n'a pas été détectée auparavant car la plupart des cas étaient asymptomatiques ou les signes présentés par les malades n'ont pas suscité la recherche diagnostique de l'infection en raison de leur manque de spécificité.

## Diagnostic et prise en charge des cas

Le dépistage des cas a bénéficié de la mise en place à Abidjan de 13 centres dédiés ainsi que de centres en provinces notamment à Bouaké et Yamoussoukro. Les prélèvements sont analysés à Abidjan au sein de l'Institut Pasteur de Côte d'Ivoire qui reçoit également les échantillons biologiques arrivant des provinces. Si au début de l’épidémie, de longs délais d'attente étaient nécessaires pour obtenir les résultats des tests, la mise en place d'une procédure de traçage et d'enregistrement, avec une plateforme automatisée de rendu des résultats, a amélioré les délais. Au 30 septembre, un total de 162 781 échantillons a été analysé par l'Institut Pasteur. Le taux de positivité a été supérieur à 20 % de la semaine 24 (8-14 juin 2020) à la semaine 29 (13-19 juillet) pour descendre sous les 10 % à partir de cette date. À partir de la semaine 38 (14-19 septembre) le taux de positivité a été inférieur à 4 % pour remonter à plus de 7 % à compter du 30 décembre avec un pic de 22 % le 12 janvier 2021 puis un autre le 18 mars (19 %), illustrant les deuxième et troisième vague. Au 17 avril 2021, un total de 572 731 examens PCR ont été réalisés en Côte d'Ivoire depuis le début de l’épidémie, et sur cette journée le taux de positivité était de 1,6 % (41 PCR positives / 2 532 tests réalisés).

La prise en charge des cas répond à la directive édictée le 4 avril 2020 par le ministère de la Santé. Celle-ci ne recommande pas l'utilisation de l'hydroxychloroquine associée à une antibiothérapie pour les cas simples mais introduit ce protocole pour les cas sévères. Les cas simples (90 % des cas actifs sont des formes bénignes) ont été isolés à domicile pour les trois-quarts d'entre eux, les autres l'ont été dans deux centres dédiés dont un complexe hôtelier réquisitionné. Les cas modérés et sévères ont été pris en charge à Abidjan au CHU de Treichville, de Yopougon et de Cocody. A la fin du mois de juillet, 150 patients diagnostiqués comme malade de la Covid-19 avaient été hospitalisés au Service de maladies infectieuses et tropicales (SMIT) du CHU de Treichville. Parmi eux, 121 avaient bénéficié d'une assistance respiratoire en réanimation et 41 décès avaient été notifiés (létalité = 27,3 % [41/150]). Depuis l'ouverture d'un service Covid-19 en mai 2020 et jusqu'en septembre, le CHU de Yopougon avait hospitalisé 84 cas de Covid-19. Parmi eux, 15 sont décédés (létalité = 17,8 % [15/84]).

La létalité globale est restée faible et inchangée depuis le début de l’épidémie. Même s'il a été supérieur à 1 % entre le 19 avril et le 7 juin (pic à 1,32 % le 19 mai), ce taux était de 0,6 % au 30 septembre 2020 (120/19 724). A cette date, cette létalité était plus importante pour la tranche d’âge de 61 ans et plus (5,3 %) avec une décroissance pour les tranches d’âge inférieures (1,1 % pour les 51-60 ans; 0,4 % pour les 41-50 ans; 0,2 % pour les 31-40 ans; 0,1 % pour les moins de 30 ans).

Sur la fin de cette première vague, au 30 septembre 2020, la Côte d'Ivoire ne comptait plus que 313 cas actifs sous surveillance médicale.

La reprise active de l’épidémie au début de l'année 2021 avec une deuxième vague en janvier puis une troisième en mars, a illustré le fait que l’épidémie continuait de progresser dans la population ivoirienne. Le nombre de cas encore actifs au 11 mars 2021 était de 3346 cas mais un mois plus tard, avec la régression de la troisième vague, ce nombre n’était plus que de 157 cas le 17 avril. Les prises en charge hospitalières sont le plus souvent concentrées au CHU de Treichville dans le service des maladies infectieuses et tropicales même si certains cas sont hospitalisés dans le secteur privé comme dans la Polyclinique Farah. Cette structure privée située dans le quartier de Marcory annonçait en fin d'année avoir accueilli plus de 500 cas de Covid-19 entre mars et octobre 2020 (communication personnelle). Les autorités sanitaires déclaraient le 17 avril 2021 que 932 personnels de santé dont 328 médecins avaient contracté l'infection à coronavirus depuis le début de l’épidémie. La létalité de 0,60 % au 17 avril 2021 est inchangée (274 décès/45 560 cas) depuis septembre 2020. Mais la tranche d’âge des plus de 60 ans enregistrait le 17 avril un taux de létalité sept fois plus élevé (4,3 % versus 0,6 %).

## Vaccination

Bénéficiant du mécanisme COVAX^2^, la Côte d'Ivoire a reçu le 26 février en provenance d'Inde un lot de 504 000 doses de vaccins Astra Zeneca produits sous licence par le *Serum Institute of India* (SII)^3^. Dès le 1^er^ mars, la campagne de vaccination a été lancée au Palais des Sports d'Abidjan. Au 15 mars, un peu plus de 15 000 doses avaient été administrées, les populations cibles prioritaires étant les personnes âgées présentant des comorbidités, les forces de sécurité et les personnels de santé. Mais une enquête réalisée en début d'année a mis en évidence la réticence des populations à se faire vacciner, avec un pourcentage de plus de 35 % de personnes réfractaires à cet acte vaccinal. Après un démarrage assez lent avec la distribution entre 1 000 et 2 000 doses par jour jusqu'au 8 avril, à partir de cette date le rythme de vaccination a augmenté passant à partir du 13 avril à environ 4 000 doses quotidiennes. Malgré l'ouverture de 61 sites de vaccination à Abidjan dont 47 dans les districts sanitaires, le 17 avril seulement 94 818 doses avaient été injectées (soit 0,35 % de la population): 83 855 premières doses et 10 963 autres pour une deuxième injection.

## Conclusion

La Côte d'Ivoire a très précocement mis en place une riposte efficace face à l’épidémie de Covid-19. Cinq jours après l'apparition du premier cas à Abidjan, des mesures contraignantes ont été édictées, notamment le confinement du Grand Abidjan qui a duré quatre mois jusqu'au 15 juillet 2020. En limitant la diffusion de l’épidémie en dehors de la capitale économique ivoirienne, les autorités sanitaires ont pu en contrôler les conséquences en provinces. Néanmoins, ces données épidémiologiques liées à la qualité de la notification des cas sont à relativiser en raison de l'accès limité au test PCR de diagnostic et à la circulation virale chez des patients asymptomatiques ou paucisymptomatiques. Par contre, la létalité enregistrée depuis le mois de mars 2020 est restée faible, inférieure à 1 %, illustrant à la fois la limitation de la mortalité du coronavirus dans une population majoritairement très jeune, et sans doute aussi la qualité du système de santé ivoirien dans sa capacité de prise en charge des cas graves. La circulation endémique d'autres coronavirus humains et la possibilité d'immunité croisée peut constituer également une des raisons du faible niveau d'incidence en Côte d'Ivoire.

À l'issue de cette crise sanitaire, il faudra néanmoins mesurer l'impact socio-économique de l’épidémie qui, bien que mieux contrôlée à la fin du mois d'avril, n'est pas terminée. L’élan des partenaires techniques et financiers, des organismes internationaux comme du secteur privé aura montré la capacité des autorités à fédérer autour d'elles pour contrer les effets de la Covid-19.

La résilience de la population ivoirienne face à l’épidémie de Covid-19 a été importante depuis un an. Hormis la période de 4 mois durant laquelle le Grand Abidjan a été confiné entre mars et juillet 2020, le desserrement des mesures contraignantes a permis aux populations et aux entreprises de reprendre presque normalement leurs activités. La possible diffusion en Côte d'Ivoire de variants d'Afrique du Sud sinon brésiliens et britanniques font également peser une menace supplémentaire sur la reprise épidémique plus forte. Les espoirs portés sur l'arrivée des vaccins doivent être tempérés par la réticence d'une partie de la population à adhérer à la campagne nationale de vaccination ainsi que par la possibilité de disposer de doses en nombre suffisant pour couvrir toute la population par une immunisation vaccinale. Les doutes nés en Europe et en Amérique du Nord sur l'innocuité du vaccin Astra Zeneca suite aux accidents thrombo-emboliques survenus chez des personnes vaccinées par ce type de vaccin ne feront qu'ajouter à la méfiance des populations ivoiriennes qui sont bien informées de l'actualité internationale, les réseaux sociaux étant très actifs dans la capitale économique qu'est Abidjan.

## Conflits D'intérêts

Les auteurs remercient Cyril Pervilhac pour sa lecture critique et les commentaires apportés au manuscrit initial.

Les auteurs ne déclarent aucun conflit d'intérêt.
